# Embryonic-stem-cell-derived mesenchymal stem cells relieve experimental contact urticaria by regulating the functions of mast cells and T cells

**DOI:** 10.1038/s41598-023-50258-2

**Published:** 2023-12-20

**Authors:** Seung Yeun Hyun, Eun-Young Kim, Minseong Kang, Jeong Won Park, Ki-Sung Hong, Hyung-Min Chung, Wahn Soo Choi, Se-Pill Park, Geunwoong Noh, Hyuk Soon Kim

**Affiliations:** 1 Department of Biomedical Sciences, College of Natural Science and Department of Health Sciences, The Graduate School of Dong-A University, Busan, 49315 Korea; 2 Mirae Cell Bio Co., Ltd., Seoul, 04795 Korea; 3 School of Medicine, Konkuk University, Seoul, 05029 Korea; 4 Department of Bio Medical Informatics, College of Applied Life Sciences, Jeju National University, Jeju, 63243 Korea; 5 Department of Allergy, Allergy and Clinical Immunology Center, Cheju Halla General Hospital, Jeju, 63127 Korea

**Keywords:** Mesenchymal stem cells, Immunosuppression, Allergy

## Abstract

Contact urticaria (CU) is an inflammatory skin disorder triggered by specific substances upon skin contact, leading to immediate acute or chronic manifestations characterized by swelling and redness. While mesenchymal stem cells (MSCs) are increasingly recognized for their therapeutic potential in immune diseases, research on the efficacy and mechanisms of stem cell therapy for urticaria remains scarce. This study investigates the regulatory role of embryonic-stem-cell-derived multipotent MSCs (M-MSCs) administered in a CU mouse model. Therapeutic effects of M-MSC administration were assessed in a Trimellitic anhydride-induced contact urticaria model, revealing significant inhibition of urticarial reactions, including ear swelling, itchiness, and skin lesion. Moreover, M-MSC administration exerted control over effector T cell activities in major lymphoid and peripheral tissues, while also suppressing mast cell degranulation in peripheral tissues. Notably, the inhibitory effects mediated by M-MSCs were found to be TGF-β-dependent. Our study demonstrates the capacity of M-MSCs to regulate contact urticaria in a murine model, harmonizing the activation of inflammatory T cells and mast cells. Additionally, we suggest that TGF-β derived from M-MSCs could play a pivotal role as an inhibitory mechanism in contact urticaria.

## Introduction

Contact urticaria (CU) is known to be a typical chronic inflammatory skin disease that causes itching, redness, swelling, and cracked skin lesions^1^. CU can be classified into two types: non-immune contact urticaria (NICU) and immune contact urticaria (ICU), a rash and flare-up reaction caused by direct contact with a symptomatic chemical or protein substance^2^. Unlike NICU, which is not dependent on a specific antibody to the causative agent, ICU involves antigens binding to the immunoglobulin E (IgE)-specific antibodies of mast cells located in the dermis^3,4^. Antigen–antibody binding, which can cause skin diseases, can also induce conditions such as degranulation of the mast cells in the skin, and vasoactive mediators such as histamine, prostaglandins, and leukotrienes are released that may cause skin microcirculation disorders^5^.

Chronic urticaria can also arise as a systemic inflammatory response involving T cell-mediated cellular immune responses in addition to peripheral innate immune responses at the time of onset. These inflammatory reactions evolve into chronic and systemic lymphocyte-mediated cellular immune responses, including T cells, leading to the simultaneous activation of direct innate and cellular immune responses to antigens^6^. Recently, the involvement of these mechanisms has been reported, and it has been revealed that, especially in the chronic exacerbation of urticaria, helper T cells and cytokines derived from them can participate in these responses^3^. Furthermore, similar to many chronic allergic and autoimmune diseases, chronic urticaria conditions may exhibit a mixed increase in T_H_1 and T_H_2 responses^7,8^.

Contact urticaria reactions often spread beyond the initial contact site, progressing to generalised urticaria. This gradual progression of the symptoms is called ‘contact urticaria syndrome’^9^. ICU involves various substances and pathophysiological factors, making its mechanism unclear, highlighting the importance of accurate diagnosis and management. Contact urticaria diseases present heterogeneous symptoms, but they are commonly associated with the immune system. Antihistamines, corticosteroids, leukotriene antagonists, and immunomodulators, widely used in the treatment of allergic diseases, are currently prevalent therapies^10^. Nevertheless, these treatments primarily alleviate symptoms by temporarily suppressing inflammatory mediators and immune cells. Their extensive use over the long term is hindered by potential side effects^10–13^. Therefore, the development of alternative therapies, offering new and fundamental approaches, becomes imperative.

In recent years, stem cell therapy has been proposed as an alternative for the treatment of several previously intractable diseases^14,15^. The injected stem cells stimulate regeneration of the damaged tissues, promote cell survival, and prevent apoptosis through the paracrine factors and cytokines^16^. Among the stem cell therapeutics, mesenchymal stem cells (MSCs) are pluripotent progenitor cells derived and isolated from a wide range of adult tissues, including bone marrow, adipose tissue, periodontal and umbilical cord blood^17^. Until recently, MSCs were reported to modulate excess inflammatory responses in autoimmune- and inflammation-related diseases, such as graft-versus-host disease, inflammatory bowel disease, multiple sclerosis, sepsis, collagen-induced arthritis, and type 1 diabetes^18–23^. In addition, several studies have shown that MSCs can improve allergic conditions, such as asthma, rhinitis, and dermatitis^24–26^. However, owing to the limited proliferative capacity during ex vivo expansion, alternative sources are needed for clinical applications.

Among several sources, human embryonic stem cells have been proposed as an important source for therapeutic MSCs^27^. The embryonic stem cell lineage established from the inner cell mass of the blastocyst can differentiate into all possible cell types and can be expanded ex vivo in an immortalised manner^28^. Given these capabilities, human embryonic stem cells are an attractive cellular resource for regenerative medicine, among other sources. Recent studies have reported a method for generating pluripotent MSCs from human embryonic stem cells^29,30^, which can overcome the shortcomings of conventional MSCs through controlled differentiation for optimal safety and efficacy prior to transplantation.

A few studies that use MSCs derived from human embryonic stem cells are still available for the treatment of CU, and the mechanism of the interactions between the MSCs and host immune cells in the pathogenesis of urticaria has not been reported. Herein, we present a non-clinical study of human embryonic-stem-cell-derived multipotent mesenchymal stem cells (M-MSCs) through an animal model of trimellitic anhydride chloride (TMA)-induced contact urticaria. This work demonstrates the therapeutic efficacy of the proposed model as well as the basis for identifying the mechanism of inhibition of inflammation.

## Materials and methods

### Animals

All animal experimental procedures were approved by the Dong-A University Medical School Institutional Animal Care and Use Committee (Approval No. DIACUC-21-11) at 22.03.2021. Female BALB/c mice that were 7–8 weeks old were purchased from Orient Bio Inc. (Gyeonggi-do, korea). The classification of experimental groups involves the random assignment of mice of the same age and within a 1 g difference in body weight after the acclimatization process. The mice are then housed in a pathogen-free facility at Dong-A University (Busan, Korea). Mice were maintained at Dong-A University facility at 22 °C ± 1 °C room temperature, 40–60% humidity, on a 12 h light–dark cycle (7 a.m. to 7 p.m.), and given food and water freely, according to institutional guidelines. All experiments were performed under inhalation anesthesia with isoflurane, and mice were euthanized by CO_2_ inhalation at the end of the experiment. This study adhered to the guidelines set forth by the laboratory animal ethics committee of Dong-A University and the ARRIVE guidelines. To ensure statistical significance, 5 or more mice per group were used, and all experimental protocols were approved by the Institutional Animal Care and Use Committee (IACUC) of Dong-A University. Inhalational anesthesia using isoflurane was used to induce anesthesia when sacrificing all experimental animals.

### Induction of contact urticaria mouse model

Contact urticaria mouse model was induced according to a previously reported method^31,32^. Mice were initially sensitized by applying 100 μl of a TMA (trimellitic anhydride; 500 mg/ml, Alfa Aesar, Ward Hill, MA, USA) in acetone/olive oil (4:1, v/v) on the shaved hind flank. This sensitization process is essential for inducing an immune response to TMA. Secondary sensitizations were performed on the hind flank to reinforce the immune response. On the 7th and 10th days after the first sensitization, mice sensitized 50 μl of a TMA solution (250 mg/ml) in acetone/olive oil (4:1, v/v). On day 13 after the initial sensitization, contact urticaria (CU) was induced by challenging the ears with 25 μl of a TMA solution (100 mg/ml) dissolved in acetone/olive oil (4:1, v/v). The disease symptoms were assessed by measuring ear thickness, itching, and lesions on the skin. Particularly, skin lesions were evaluated by determining the ratio of the affected area, indicating erythema and edema on a 3 cm^2^ area of the dorsal skin. The symptom evaluation of experimental animals was evaluated based on all animals without exclusion criteria. For histological analysis, H&E and mast cell staining were conducted. Cellular and molecular analysis involved the use of flow cytometry to assess immune cell activity in mouse lymphoid organs, along with genetic analysis of the lesions. Experimental animals of 10 mice per group were performed by blindly selecting 5–6 mice for efficient handling of animal-derived flow cytometric and genetic analysis. The corresponding author and one of the first authors (S.Y. Hyun) were aware of the group assignment, and the symptoms, flow cytometry, and other molecular analysis results were evaluated together by multiple blinded co-authors.

### Magnetic-activated cell sorting (MACS)

For the purposes of in vitro experiments, we used the naïve CD3^+^ T cell isolation Kit (Miltenyi Biotec, Bergisch-Gladbach, Germany) to enrich naïve CD3^+^ cells from the spleens of BALB/c mice (6 weeks old). All steps were conducted strictly following the manufacturer’s protocol.

### Cell culture

M-MSCs used in this study was provided Mirae Cell Bio (Seoul, Korea). M-MSCs differentiated from H9 hESCs^30^ were maintained in EGM2-MV medium (Lonza, San Diego, CA, USA) containing supplement Mix (promocell, Heidelberg, Germany) and 50 ug/ml Gentamicin (Gibco, NewYork, USA) in a humidified atmosphere containing 5% CO_2_ at 37℃, as previously described^33^. M-MSCs at less than ten passages were used for in vitro cell culture and in vivo animal experiments. Bone marrow-derived MSCs (BM-MSCs) were maintained in MSCBM medium (Lonza, Basel, Switzerland) containing supplement kit (Lonza) in a humidified incubator at 5% CO_2_ at 37℃. Bone marrow-derived mast cells (BMMCs) derived from BALB/c mice were cultured in RPMI 1640 medium containing 2 mM L-glutamine, 0.1 mM nonessential amino acids, antibiotics, 10% fetal bovine serum (FBS), and IL-3 (10 ng/ml; PeproTech Inc., Rocky hill, NJ, USA). After 4 weeks, > 98% of the cells were verified as BMMCs, as previously described^34^. Mouse splenic T cells were presorted by CD3 mAb-microbeads (Miltenyi Biotec, Bergisch Gladbach, Germany) followed by the manufacturer's method. For T cell polarization, splenic naïve CD3^+^ T cells were cultured onto a 24-well plate coated with 1 μg/ml of anti-CD3 (eBioscience, San Diego, CA, USA) in complete RPMI 1640 medium and supplemented with T_H_1 reagents [IL-2 (20 ng/ml, PeproTech Inc.), IL-12 (20 ng/ml, PeproTech Inc.), and anti-IL-4 (10 μg/ml, Bio X cell, West Lebanon, NH, USA)] or T_H_2 reagents [IL-2 (20 ng/ml, PeproTech Inc.), IL-4 (100 ng/ml, PeproTech Inc.), and anti-IFN-γ (10 μg/ml, Bio X cell)]. After 48 h, the cells were co-cultured with M-MSCs for 24 h under polarized conditions. To confirm the regulation of immune cells by M-MSCs in vitro, BMMCs (5.0 × 10^5^ cells/well), splenic T cells (5.0 × 10^5^ cells/well), or polarizing splenic T cells (5.0 × 10^5^ cells/well) were co-cultured with the indicated ratio of M-MSCs for 24 h. Co-cultured splenic T cells were identified by flow cytometry analysis. The analysis of polarized T cells was assessed by distinguishing them using the gating strategy as depicted in Supplementary Fig. S1. The ratio of degranulation of BMMCs was analyzed by β-hexosaminidase secretion.

### Adoptive transfer and anti-TGF-β mAb neutralization

After the primary sensitization of the contact urticaria model, M-MSCs were injected subcutaneously into the ear as a single administration on the 10th days or twice on the 10th and 12th days. A primary disease improvement evaluation was performed through single administration or two administrations, and an appropriate cell administration group for disease efficacy was selected. The administration of an equal amount of BM-MSCs and oral administration of cetirizine (50 mg/kg) (Sigma-Aldrich, St. Louis, MO, USA) were used as positive controls. To deplete TGF-β, BALB/c mice were intraperitoneally injected with 300 μg of anti-TGF-β mAb (1D11.16.8, Bio X cell) or an isotype-matched control mAb (Bio X cell) twice on days 0 and 3 of M-MSCs administration.

### Flow cytometric analysis

Single-cell suspensions were isolated from the spleen, cervical lymph node (cLN), and ear. Ear tissues were isolated into single cells using the gentleMACS dissociator (Miltenyi Biotec) followed by the manufacturer’s method. For the detection of intracellular cytokines, the isolated cells were stimulated with PMA (50 ng/ml; Sigma-Aldrich), ionomycin (500 ng/ml; Sigma-Aldrich), and brefeldin A (3 μg/ml; eBioscience) for 4 h before analysis and a fixation/permeabilization kit were from eBioscience. Before cell surface markers were stained, Fcγ receptors were blocked with anti-CD16 and anti-CD32 mAbs (2.4G2, BD Biosciences), and conjugated and dead cells were excluded by analysis on the basis of forward and side light scatter parameters and staining with a Zombie NIR™ Fixable Viability Kit (Biolegend, San Diego, CA, USA). The antibodies against proteins were as follows: Antibodies against CD3 (17A2) and CD8a (53-6.7) were obtained from BioLegend. Antibodies for CD4 (RM4-5), IFN-γ ((XMG1.2), IL-4 (11B11) were obtained from eBioscience. Antibodies for CD3 (17A2), CD45 (30-F11), and CD127 (A7R34) were obtained from BioLegend. The cells were then analyzed with a NovoCyte flow cytometer (Agilent) and FlowJo version 10 software (Tree Star, Ashland, OR, USA).

### β-hexosaminidase assay

BMMCs (5.0 × 10^5^ cells/well) co-cultured with M-MSCs (0.5 to 2 × 10^6^ cells/well) for 24 h were sensitized for 4 h with Monoclonal dinitrophenol (DNP)-specific IgE (100 ng/ml; Sigma). The IgE-primed BMMCs were then stimulated with 50 ng/ml of DNP-human serum albumin (DNP-HSA, Sigma-Aldrich) in Tyrode-BSA buffer (20 mM Hepes (pH 7.4), 135 mM NaCl, 5 mM KCl, 1.8 mM CaCl_2_, 1 mM MgCl_2_, 5.6 mM glucose, and 0.1% BSA) for 15 min in the presence or absence of the M-MSCs.

Degranulation was determined by measuring the release of the granule marker β-hexosaminidase as previously described^35^. The degree of degranulation of BMMCs was expressed as the % of the activity of β-hexosaminidase secreted out of the cells compared to the total activity of β-hexosaminidase.

### Reverse transcriptase (RT)-PCR and quantitative Real-time PCR

M-MSCs were co-cultured with splenic T cells or BMMCs for 24 h and then effector cells were removed. M-MSCs were rinsed with PBS and left on ice for 5 min to stop the reaction. Total RNA was extracted using AccuPrep® Universal RNA Extraction Kit (Bioneer, Daejeon, Korea), and cDNA was synthesized using AccuPower® CycleScript RT PreMix (Bioneer) according to the manufacturer’s instructions. The PCR reaction was amplified using AccuPower® PCR PreMix (Bioneer) and PCR was performed at 95 ℃ for 2 min, 95 ℃ for 20 s, 58 ℃ for 40 s, 72 ℃ for 30 s, 72 ℃ for 5 min for 30 cycles. Primers used as follow: human *Hgf* (forward 5′-TCCATGATACCACACGAACACAGC-3′, reverse 5′-TGCACAGTACTCCCAGCGGGTGTG-3′); human *Ido1* (forward 5′-TTTGCTAAAGGCGCTGTTGG-3′, reverse 5′-CCTTCATACACCAGACCGTCTGA-3′); human *Pdl1* (forward 5′-TATGGTGGTGCCGACTACAA-3′, reverse 5′-TGCTTGTCCAGATGACTTCG-3′); human *Il10* (forward 5′-AGACATCAGGGTGGCGACTCTAT-3′, reverse 5′-GGCTCCCTGGTTTCTCTTCCTAAG-3′); human *Pge2* (forward 5′-ACCATCTACCCCTTCCTTT-3′, reverse 5′-CCGCTTCCCAGAGGATCT-3′); human *Tgfb* (forward 5′-GGGACTATCCACCTGCAAGA -3’, reverse 5′-CCTCTTGGCGTAGTAGTCG-3′); human *Gapdh* (forward 5′-ACCACAGTCCATGCCATCAC-3′, reverse 5′-TCCACCACCCTGTTGCTGTA-3′). Snap-frozen disease-inducing mouse ear tissues were ground to powder. Total RNA isolation and PCR reaction were performed in the same manner as above. Real-time PCR was performed Thermal Cycler Dice^Ⓡ^ Real Time System III TP950 (Takara, Shiga-ken, Japan). Primers used as follow: mouse *Il4* (forward 5′-ACAGGAGAAGGGACGCCAT-3′, reverse 5′-GAAGCCCTACAGACGAGCTCA-3′); mouse *Il6* (forward 5′-GAGGATACCACTCCCAACAGACC-3′, reverse 5′-AAGTGCATCATCGTTGTTCATACA-3′); mouse *Ifng* (forward 5′-CAGCAACAGCAAGGCGAAAAAGG-3′, reverse 5′-TTTCCGCTTCCTGAGGCTGGAT-3′); mouse *Tnfa* (forward 5′-AGTGACAAGCCTGTAGCCCACGT -3′, reverse 5′-CCATCGGCTGGCACCACTAGTT-3′); mouse *Gapdh* (forward 5′-CATCACTGCCACCCAGAAGACTG-3′, reverse 5′-ATGCCAGTGAGCTTCCCGTTCAG-3′);

### Histological analysis

After the induction of contact urticaria in mice, their ear tissues were fixed in 4% paraformaldehyde in phosphate-buffered saline for 24 h and then embedded in paraffin. The tissues were dehydrated in a graded ethanol series (70 to 100%), rinsed three times with xylene for 3 min each, and then embedded in paraffin. Sections of paraffin-embedded tissues, with a thickness of 6 μm, were prepared and stained with hematoxylin (Sigma-Aldrich) and eosin (Sigma-Aldrich) to compare and analyze the degree of cell invasion and epidermal thickness in the tissue. Additionally, sections of tissues with a thickness of 6 μm were stained with a 1% toluidine blue (Sigma-Aldrich) solution to assess the number of infiltrating mast cells and the degree of degranulation.

### Statistical analysis

The in vitro experiment was repeated three independent times, and the animal experiment was based on five or more animals per group, and if the results of the first experiment were insufficient, the significance was evaluated within a total of 10 animals in the group. The data are presented as the mean ± standard error (SEM) from three or more independent experiments for in vitro experiments. Statistical analysis was done by unpaired Student's *t*-test. One-way analysis of variance (ANOVA) with Tukey's post hoc test was performed for multiple comparisons. Statistical significance (*P < 0.05 and **P < 0.01) was determined with Prism version 7.0 (GraphPad, San Diego, CA).

### Ethics approval and consent to participate

This study was approved by the Institutional Animal Care and Use Committee (IACUC) of Dong-A University (DIACUC-21-11). All animal experiments were performed in accordance with the guidelines and regulations of the institutional guidelines.

## Results

### Inhibitory effects of M-MSC administration in contact urticaria mouse model

**Figure 1:**
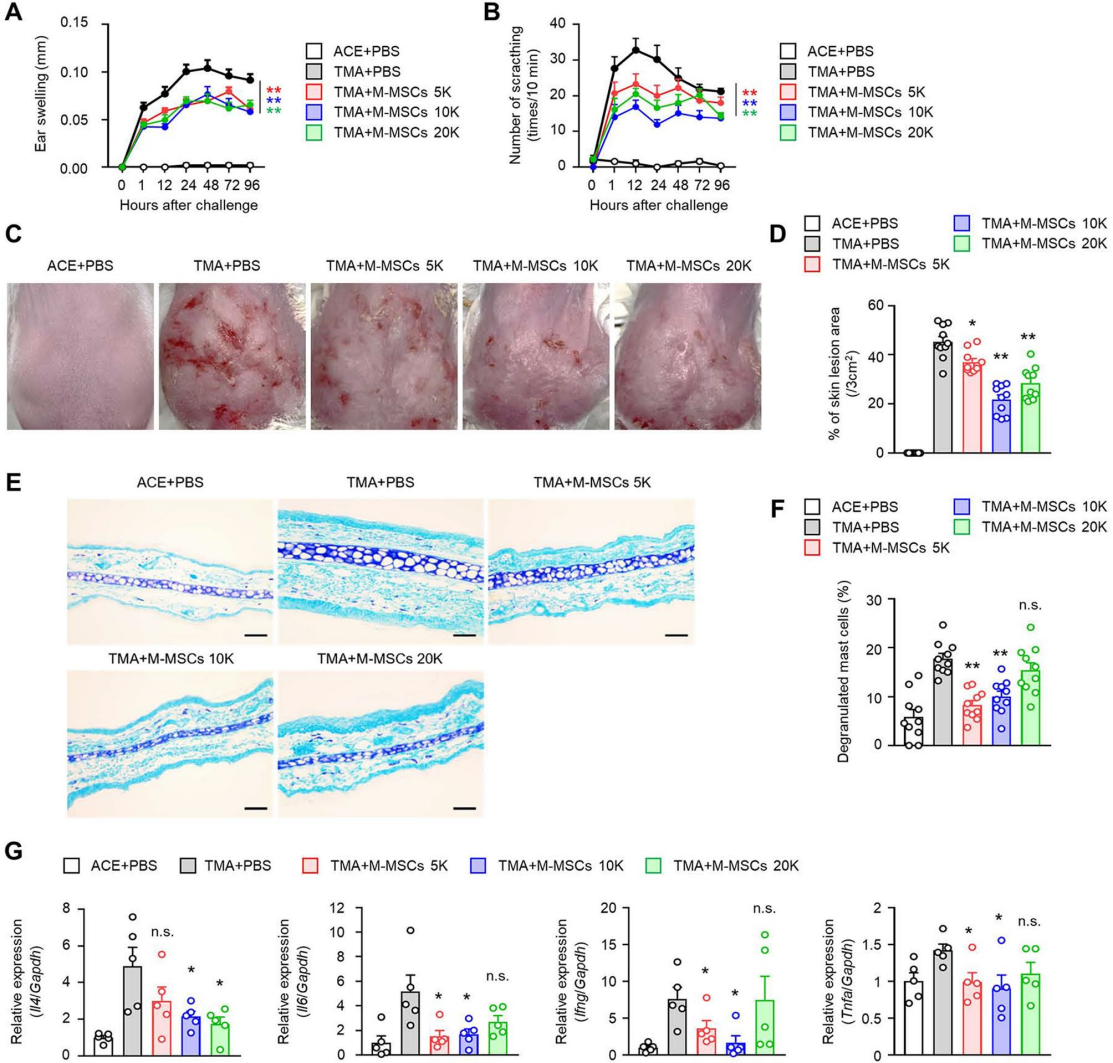
M-MSCs attenuate CU in vivo. Data for the ear thickness (**A**), scratching behaviour (**B**), and % of skin lesion area (**C**,**D**) in TMA-induced CU mice with or without transfer of M-MSCs (0.5 to 2.0 × 10^4^). **E** Degranulation of mast cells (MCs) was examined by toluidine blue stain (scale bar: 100 µm), and (**F**) the histograms show the ratio of degranulated MCs in ear tissues. The results are expressed as mean ± SEM (**A**–**F**) (*n* = 10). *p < 0.05, **p < 0.01; n.s., not significant versus TMA + PBS group by Student’s *t* test. (**G**) Relative mRNA expression levels of *Il4, Il6, Ifng,* and *Tnfa* were determined (*n* = 5). The results are expressed as mean ± SEM of five independent experiments. *p < 0.05, **p < 0.01; n.s., not significant versus TMA + PBS group by Student’s *t* test.

The administration of MSCs has been reported to have therapeutic effects in various mouse models of dermatitis, but it was not known whether the human embryonic stem cell-derived M-MSCs we propose play a complementary role in CU. We evaluated the effects of M-MSC administration at various concentrations in the CU model and observed an overall reduction in clinical indicators such as ear swelling (Fig. 1A), itching (Fig. 1B), and skin lesion areas (Fig. 1C,D) in the M-MSC treatment group compared to the control group. Although a dose-dependent relief effect was not confirmed, the M-MSCs 10 K group was identified as the most significant condition for disease improvement. Subsequently, we examined the influence of M-MSC administration on the activation of mast cells in the ear tissue. It was confirmed that the administration of M-MSCs at 5 K and 10 K significantly reduced the number of degranulated mast cells in the tissue (Fig. 1E,F). To assess whether inflammatory cytokines are regulated in ear tissue, we analyzed the mRNA expression control effects of representative inflammatory cytokines such as Interleukin (IL)-4, IL-6, Interferon (IFN)-γ, and Tumor necrosis factor (TNF)-α. Similar to the activation of mast cells in the ear, mRNA expression of inflammatory cytokines in peripheral ear tissue was most significantly inhibited in the M-MSCs 5 K and 10 K transferred groups, but the M-MSCs 20 K group showed inconsistent effects on the reduction (Fig. 1G).

**Figure 2:**
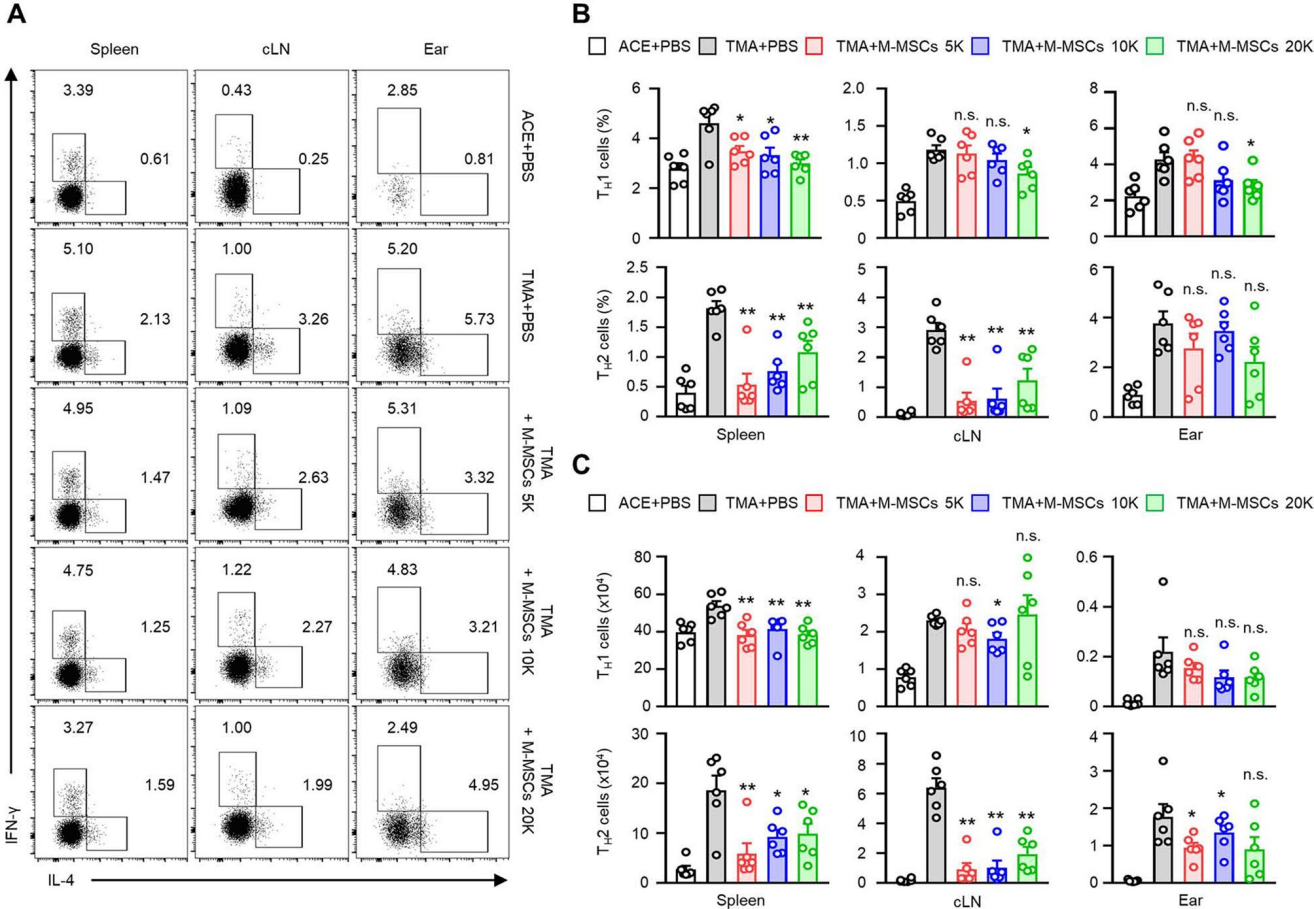
M-MSCs modulates the activity of T cells. (**A**) Representative images showing the T_H_1, T_H_2 cells in the spleen, cLN, and ear from the TMA mice. Histograms showing frequencies (**B**) and numbers (**C**) of T_H_1, T_H_2 cells for panel (**A**) (*n* = 6). The results are summarized in the bar graphs, and the data are expressed as mean ± SEM. *p < 0.05, **p < 0.01; n.s., not significant versus TMA + PBS group by Student’s *t* test.

The CU disease model employed in this study represents a systemic inflammatory condition wherein T cell-mediated immune responses are demonstrated through prior antigen exposure and re-recognition of the antigen^2^. Therefore, it is necessary to analyse the changes in the activities of the T cells as major effectors in the inflammatory response. Ninety-six hours after induction of contact urticaria, the mice were euthanised, and single cells were extracted from the spleen, cervical lymph nodes (cLNs), and ear tissues to evaluate the activities of the helper T cells. Compared to the PBS-administered control group, all M-MSCs treated groups exhibited a significant reduction in the frequency and count of IFN-γ^+^ T_H_1 cells within the spleen. Within the same batch, a decrease in the frequency and count of IL-4^+^ T_H_2 cells in the cLN was observed in all M-MSCs treated groups, In the targeted area of the disease, the ear, there was no significant overall difference in the frequency trend. However, notably, the increased number of Th2 cells with disease development was significantly suppressed in the M-MSCs 5 K and 10 K treatment groups (Fig. 2). Considered together, this suggests that the subcutaneous administration of M-MSCs produces a disease inhibitory effect in mice with CU.

### Evaluation of changes in contact urticaria improvement by repeated administration of M-MSCs

**Figure 3:**
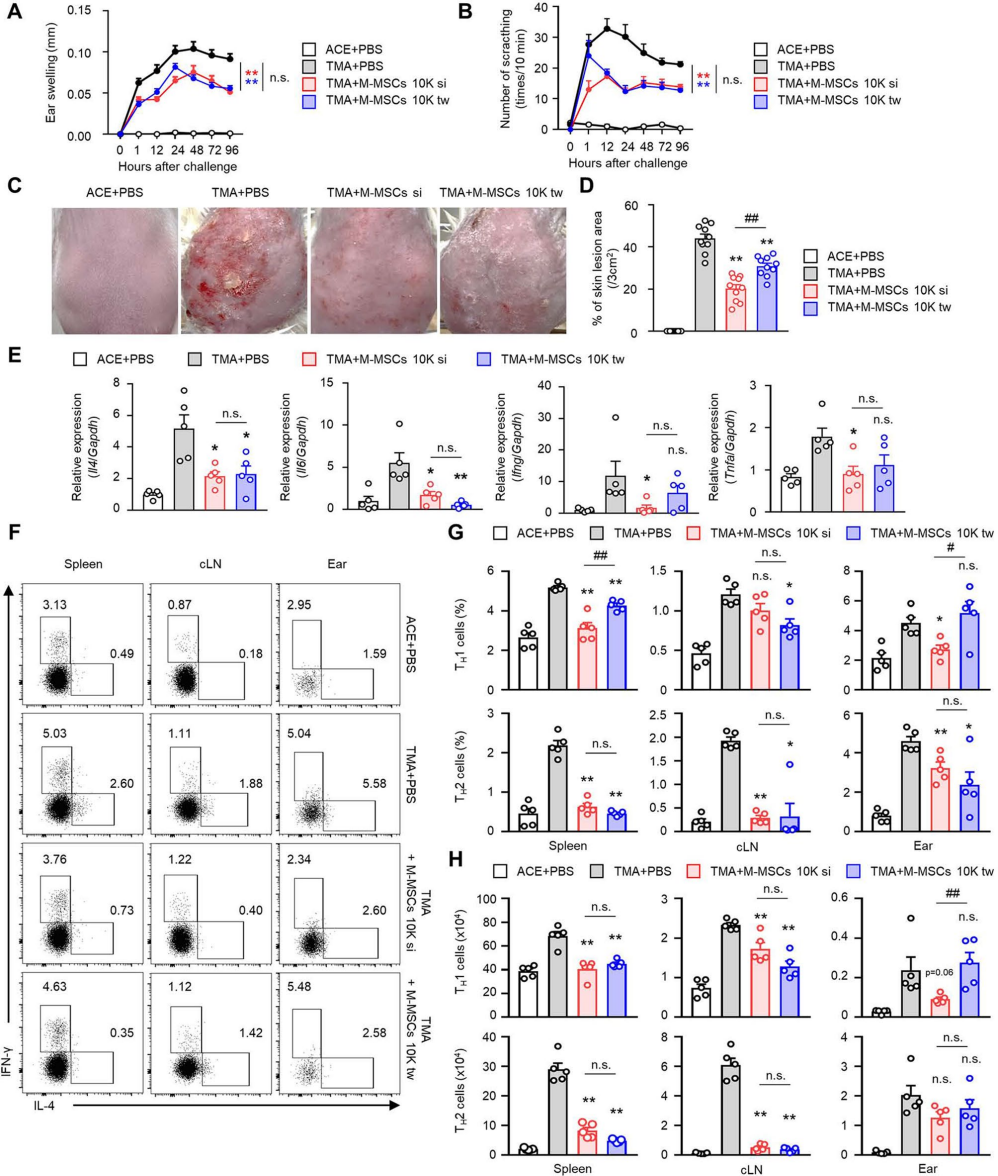
Evaluation of urticaria amelioration through repeated administration of M-MSCs. Data for the ear thickness (**A**), scratching behaviour (**B**), and % of skin lesion area (**C**,**D**) in TMA-induced CU mice with single (si) and repeated (twice; tw) administrations of adoptive transferred M-MSCs. The results are expressed as mean ± SEM (**A**–**F**) (*n* = 10). *p < 0.05, **p < 0.01; n.s., not significant versus TMA + PBS group and ^##^p < 0.01 versus TMA + M-MSCs 10 k si by one-way ANOVA with post-hoc Tukey’s test. **E** Relative mRNA expression levels of *Il4, Il6, Ifng,* and *Tnfa* were determined. The results are expressed as mean ± SEM of five independent experiments (n = 5). *p < 0.05, **p < 0.01; n.s., not significant versus TMA + PBS group by Student’s *t* test. (**F**) Representative images showing T_H_1, T_H_2 cells in the spleen, cLN, and ear from TMA mice. Histograms showing frequencies (**G**) and numbers (**H**) of T_H_1, T_H_2 cells for panel (**F**) (*n* = 5). The data are expressed as mean ± SEM. *p < 0.05, **p < 0.01; n.s., not significant versus TMA + PBS group and ^#^*p* < 0.05, ^##^*p* < 0.01 versus TMA + M-MSCs 10 k si by one-way ANOVA with post-hoc Tukey’s test.

In contact urticaria skin disease in vivo, the therapeutic effects of M-MSC administration were confirmed, and the improvements were observed even with administration of a small number of M-MSCs. Based on the results in Fig. 1, the group with a single administration of 1 × 10^4^ M-MSCs exhibited the most significant improvement in contact urticaria. It was evaluated whether synergistic effects could be achieved through repeated administration of M-MSCs. In the method of repeated administration, the same amount of M-MSCs was administered to the same lesion 2 days after the initial single administration to confirm each major disease parameter. Similar to previous results, comparing the negative control group that was administered PBS, the trends with regard to suppression of ear edema (Fig. 3A), alleviation of itching (Fig. 3B), and reduction of the area of skin lesion (Fig. 3C,D) were confirmed through all of M-MSC administration. These conditions showed significant inhibitory effects in both the single (si) and repeated (twice; tw) M-MSC administration groups. Thus, additional administrations did not yield a significant effect in suppressing the disease.

Although it was not possible to distinguish specific differences in the external lesions, the expressions of inflammatory factors in the tissues according to the M-MSC administration method were compared. In the single administration group, significant inhibitory effects were confirmed for all inflammatory cytokines, but in the repeated administration group, inhibitory effects were confirmed only for IL-4 and IL-6 (Fig. 3E). In addition, in terms of modulation of the T cell activity, the distribution and cell number inhibitory effects of each M-MSC administration group compared to the PBS group were confirmed (Fig. 3F–H). This analysis of the inhibitory effect confirmed that there was no dose-dependent synergy effect of M-MSCs. A single administration of M-MSCs 10 K was sufficient to observe an inhibitory effect.

### Comparison between administration of M-MSCs and positive controls

**Figure 4:**
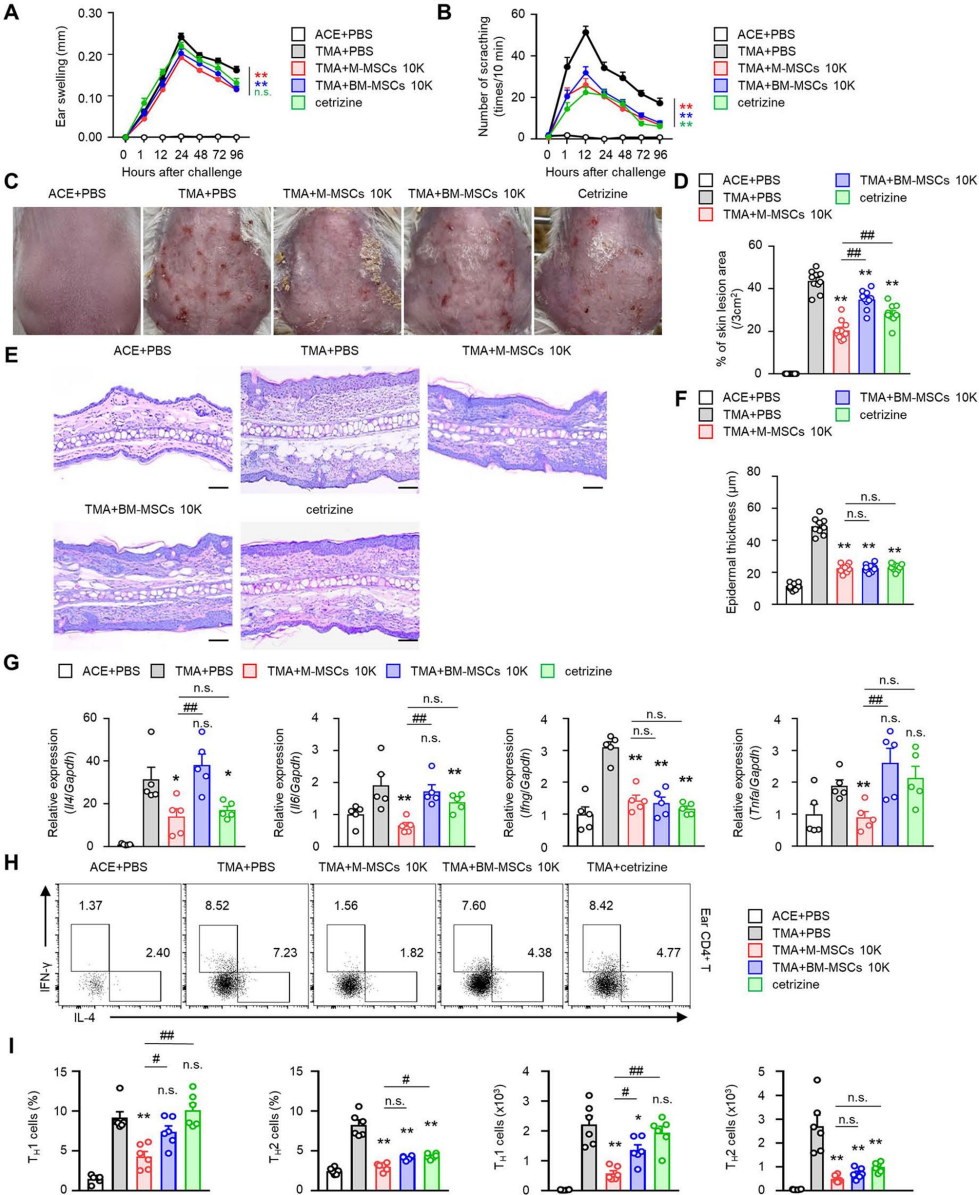
M-MSCs effectively suppress more CU responses than BM-MSCs. Data for ear thickness (**A**), scratching behaviour (**B**), and % of skin lesion area (**C**,**D**) in TMA-induced CU mice with transfer of M-MSCs or BM-MSCs or cetirizine. (**E**) Representative H&E images (4 days after TMA challenge) of the ear tissues (scale bars: 100 µm), and (**F**) histograms of ear epidermal thickness The results are expressed as mean ± SEM (**A**–**F**) (n = 10). *p < 0.05, **p < 0.01; n.s., not significant versus TMA + PBS group by Student’s *t* test. ^##^*p* < 0.01 versus TMA + M-MSC 10 k by one-way ANOVA with post-hoc Tukey’s test. **G** Relative mRNA expression levels of *Il4, Il6, Ifng,* and *Tnfa* were determined (*n* = 5). The results are expressed as mean ± SEM of five independent experiments. *p < 0.05, **p < 0.01; n.s., not significant versus TMA + PBS group by Student’s *t* test. ^##^*p* < 0.01 versus TMA + M-MSC 10 k by one-way ANOVA with post-hoc Tukey’s test. **H** Representative images showing T_H_1, T_H_2 cells in the ear tissue from TMA mice (*n* = 6). Histograms showing frequencies (**I**) and numbers (**J**) of T_H_1, T_H_2 cells for panel (**H**). The data are expressed as mean ± SEM. *p < 0.05, **p < 0.01; n.s., not significant versus TMA + PBS group by Student’s *t* test. ^#^*p* < 0.05, ^##^*p* < 0.01 versus TMA + M-MSCs 10 k by one-way ANOVA with post-hoc Tukey’s test.

Human bone-marrow-derived mesenchymal stem cells (BM-MSCs) have been reported to be effective in various inflammatory disease models^36–45^, and the interactions between the BM-MSCs and T cells are well established^43–46^. Therefore, we evaluated the effectiveness of M-MSCs together with BM-MSCs as a positive control for M-MSCs and cetirizine, a second-generation antihistamine commonly used to treat allergic rhinitis, dermatitis, and urticaria. First, the effects of suppressing edema and itching of the ear were confirmed, and the M-MSCs-administered group showed similar inhibitory effects as the BM-MSC-administered group (Fig. 4A,B). In terms of reduction of the area of skin lesion, significant inhibitory effects were observed in all administration groups, but the M-MSC-administered group showed improved inhibitory effects than the positive control groups (Fig. 4C,D). Next, we analyzed the thickness change of the epidermis through histological analysis of the ear. We confirmed a reduction in epidermal thickness for all groups administered with M-MSCs, BM-MSCs, and cetirizine, compared to the disease-induced PBS administration group. However, better inhibitory effects were not significantly noted in the comparison between the groups that were administered M-MSCs and positive control (Fig. 4E,F). However, the inhibitory effects of the M-MSC-administered group were more effective than those of the positive controls on the expression of peripheral inflammatory cytokines. Except for the expression of IFN-γ, we confirmed that inhibition of the mRNA expressions of IL-4, IL-6, and TNF-α was significantly more effective than those in the BM-MSC-administered group (Fig. 4G). In the contact urticaria in vivo model, the effects of M-MSC administration were confirmed to be more effective than the other positive controls, particularly in terms of inhibition of T_H_1 cell frequency and number in the peripheral tissues (Fig. 4H,I).

### Effects of inhibiting T cell and mast cell activities by M-MSCs in vitro

**Figure 5:**
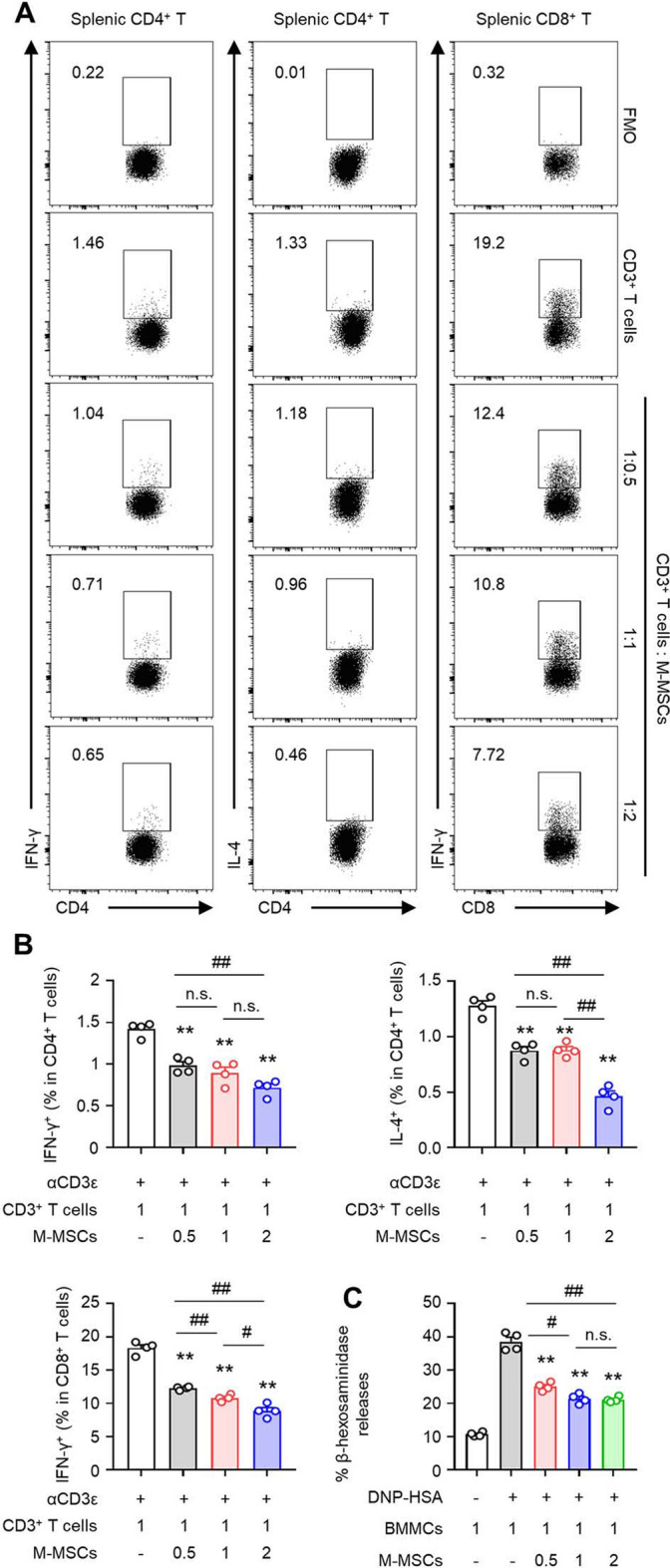
M-MSCs suppress activation of T cells and MCs. (**A**,**B**) Different ratios of M-MSCs and splenic T cells were co-cultured for 24 h with αCD3 (1 µg/ml), and the (**A**) representative images and (**B**) histograms for flow cytometry analysis of T_H_1, T_H_2, and Tc cells (*n* = 4). (**C**) Different ratios of M-MSCs and BMMCs and were cocultured for 24 h, and β-hexosaminidase release was detected (*n* = 4). The values represent the mean ± SEM from four independent experiments. *p < 0.05, **p < 0.01; n.s., not significant by Student’s *t* test.

We confirmed the inhibitory effects of M-MSC administration in an in vivo contact urticaria model, and additionally, we evaluated whether M-MSCs directly regulate the activities of major effector cells, T cells, and mast cells. We confirmed that the control effects of co-cultured M-MSCs on pro-inflammatory cells could be reproduced under activation of splenic-derived T cells and bone-marrow-derived mast cells. To determine whether the M-MSCs exert a regulatory effect on the pro-inflammatory cells in vitro by co-culture of the spleen-derived T cells and bone-marrow-derived mast cells with the indicated dose of M-MSCs, we confirmed that the activities of the T_H_1, T_H_2, and cytotoxic T (Tc) cells were inhibited in a ratio-dependent manner by the co-cultured M-MSCs under the stimulation of the TCR signalling mechanism of the isolated T cells (Fig. 5A,B). The mast cells, another type of effector cells in contact urticaria, demonstrated inhibited degranulation in a ratio-dependent manner when co-cultured with M-MSCs under the stimulation of the IgE-FcεRI signaling mechanism (Fig. 5C).

### TGF-β gene expression of M-MSCs by interactions with effector cells

From the above in vitro results, we observe that M-MSCs control the activation of effector lymphocytes and degranulation of mast cells. Therefore, we tried to determine the regulatory molecules of the M-MSCs that block the functions of the effector cells. The mRNA expressions of the hepatocyte growth factor (HGF), indoleamine 2,3-dioxygenase-1 (IDO-1), programmed death-1 ligand (PD-L1), IL-10, prostaglandin-E2 (PGE2), and transforming growth factor-β (TGF-β, a known representative human MSC-derived regulator) were analysed under co-culture conditions^47^.

**Figure 6:**
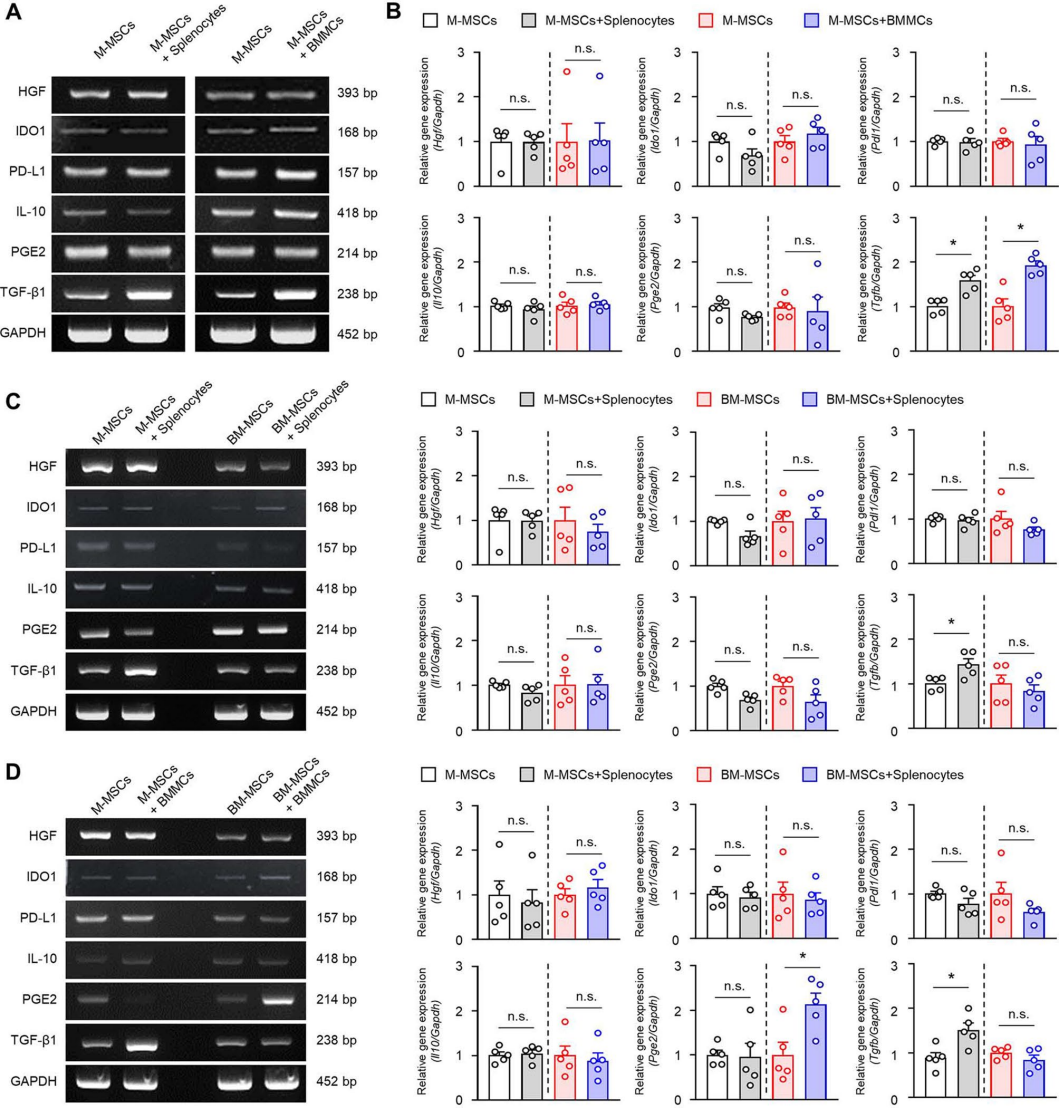
TGF-β gene expression induced by M-MSCs. (**A**,**B**) M-MSCs were co-cultured with splenocytes or BMMCs for 24 h. The levels of human *Hgf, Ido1, Pdl1, Il10, Pge2,* and *Tgfb* in the M-MSCs were examined by RT-PCR. *Gapdh* was used as the control. Original gels are presented in Supplementary Fig. S2. Significant differences against the M-MSCs, *p < 0.05; n.s., not significant by Student’s *t* test. (*n* = 5). (**C**,**D**) M-MSCs or BM-MSCs were co-cultured with splenic T cells or BMMCs for 24 h. The human *Hgf, Ido1, Pdl1, Il10, Pge2,* and *Tgfb* mRNA were measured using RT-PCR. Original gels are presented in Supplementary Figs. S3 and S4. Representative images and graphs are shown as mean ± SEM from five independent experiments. Significant differences against the M-MSCs or BM-MSCs, *p < 0.05, n.s., not significant by Student’s *t* test (*n* = 5).

After 24 h of co-culture, when the above human M-MSC-derived mRNA expressions were confirmed, the gene expression of the M-MSC-derived TGF-β was observed under co-culture with splenocytes and mast cells (Fig. 6A,B, original gels are presented in Supplementary Fig. S2). We confirmed that there was a significant increase compared to the M-MSCs alone. When BM-MSCs, which are MSCs from other sources, were co-cultured with the effector cells, there was no significant increase in the TGF-β mRNA, unlike that with the M-MSCs (Fig. 6C,D, original gels are presented in Supplementary Figs. S3 and S4). This suggests that TGF-β derived from M-MSCs may play a crucial role in regulating the activity of effector cells in contact urticaria.

### In vitro TGF-β signalling-dependent inhibitory effect on effector cells by M-MSCs

**Figure 7:**
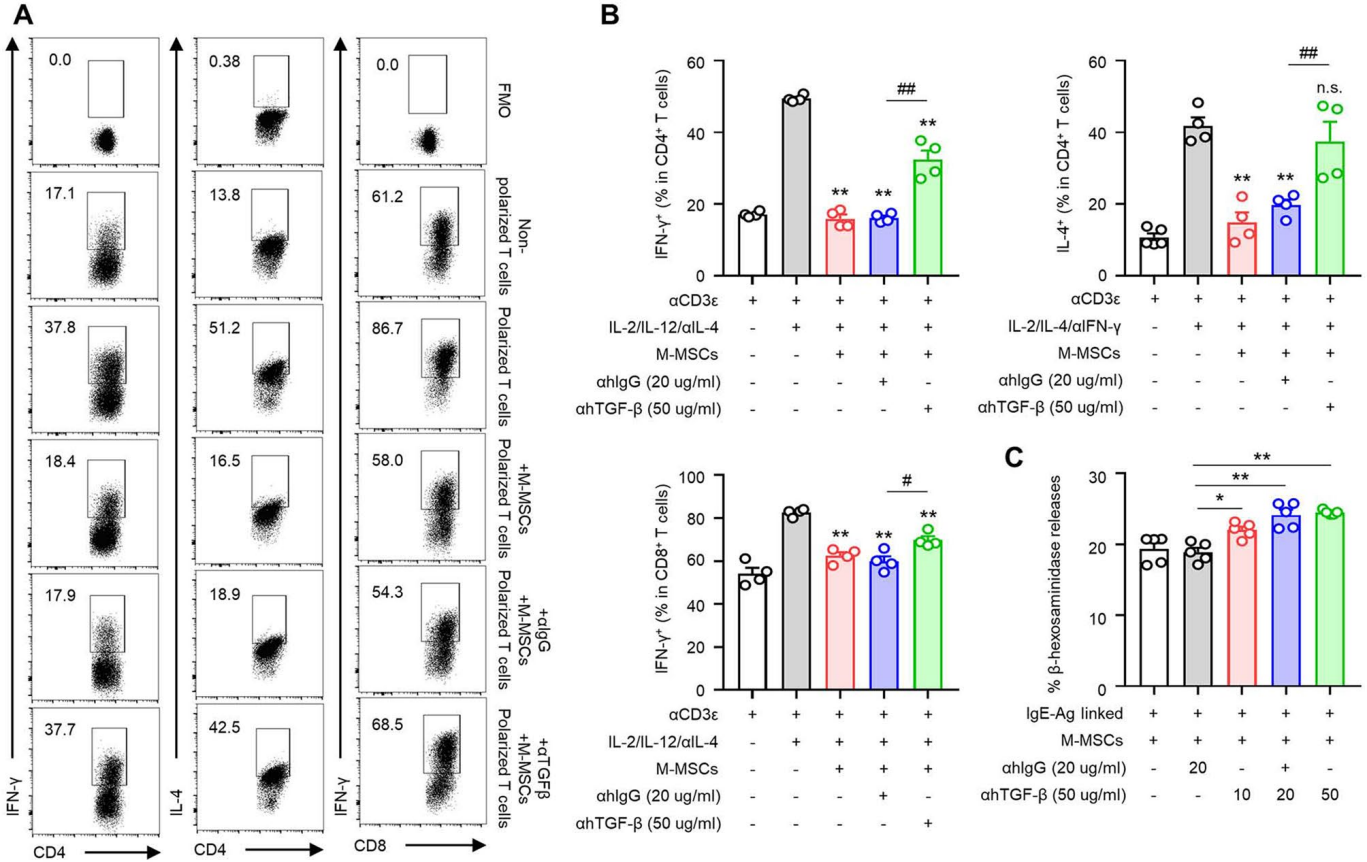
M-MSCs suppress T cells and MCs based on TGF-β. (**A**) Representative flow cytometry images and (**B**) histograms for the frequency of polarized T cells (*n* = 4). *p* < 0.05, **p < 0.01; n.s., not significant versus non-cocultured T cell group by Student’s *t* test. ^#^*p* < 0.05, ^##^*p* < 0.01; n.s., not significant versus M-MSCs + αhIgG group by one-way ANOVA with post-hoc Tukey’s test. (**C**) IgE-Ag linked MCs and M-MSCs were cocultured for 24 h and β-hexosaminidase release was measured. The values represent the mean ± SEM from five independent experiments. *p < 0.05, **p < 0.01 versus M-MSCs + αhIgG group by Student’s *t* test.

We used in vitro TGF-β monoclonal antibodies (mAb) to neutralize the M-MSC-mediated TGF-β mechanism. In the condition that M-MSCs were co-cultured with polarised T cells and mast cells for 24 h, TGF-β mAb was treated to confirm whether the TGF-β-dependent mechanism was important. When M-MSCs and effector T cells were co-cultured, it was confirmed that the suppressed activities of T_H_1, T_H_2, and T_c_ cells could be partially recovered with TGF-β mAb (Fig. 7A,B). Additionally, when co-cultured with M-MSCs and FcεRI signaling-activated mast cells, we observed that the suppression of mast cell degranulation by M-MSCs was restored in a dose-dependent manner upon treatment with a TGF-β neutralizing antibody (Fig. 7C). Although various immunomodulatory factors are derived from M-MSCs, we confirmed that M-MSC-derived TGF-β could significantly control the functions of the effector cells in contact urticaria.

### Effects of TGF-β-mediated M-MSC administration in contact urticaria disease model

**Figure 8:**
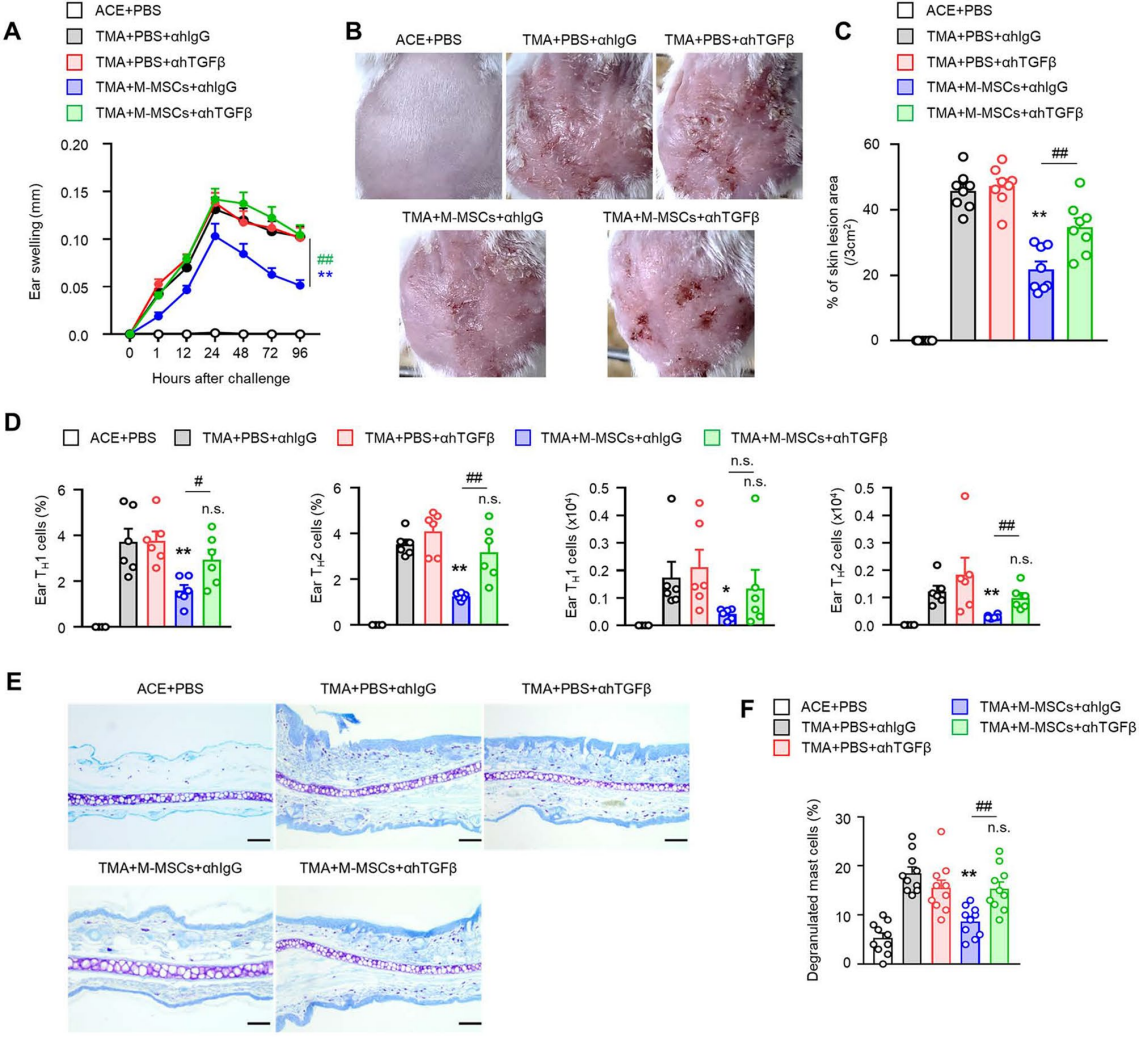
Inhibition of CU by M-MSCs through TGF-β. Data for ear thickness (**A**) and % of skin lesion area (**B**,**C**) in TMA-induced CU mice with or without transfer of M-MSCs and anti-hTGF-β mAb (300 μg i.p.) (n = 8). **D** The histograms show the frequencies and numbers of T_H_1, T_H_2 cells in the ear tissue from CU mice (*n* = 6). (**E**) Degranulation of mast cells (MCs) was examined by toluidine blue stain (scale bar: 100 µm), and (**F**) the histograms show the ratio of degranulated MCs in ear tissues. The data are expressed as mean ± SEM. *p < 0.05, **p < 0.01; n.s., not significant versus TMA + PBS + αhIgG group by Student’s *t* test. ^#^*p* < 0.05, ^##^*p* < 0.01; n.s., not significant versus TMA + M-MSCs + αhIgG group by one-way ANOVA with post-hoc Tukey’s test.

Through our previous results, we confirmed the therapeutic effects of subcutaneously administered M-MSCs in the CU disease model. Additionally, we observed changes in M-MSC-derived TGF-β gene expression and confirmed that the inhibitory effects of M-MSC were limited by TGF-β mAb neutralization in co-culture with effector cells in vitro. Therefore, to further evaluate whether TGF-β is involved in the inhibitory effects of M-MSCs on contact urticaria, we examined the alterations in the inhibitory effects exerted by M-MSCs through in vivo TGF-β mAb treatment for neutralization. In the TMA-induced CU disease model, we observed that the inhibitory effects on ear swelling (Fig. 8A) and skin lesions (Fig. 8B,C) caused by the administration of M-MSCs were exacerbated by the administration of a TGF-β neutralizing antibody. It was also confirmed whether the inhibitory effects of M-MSC administration by TGF-β neutralising antibody treatment affected the activities of the effector T cells in the ear. Treatment with the TGF-β-neutralising antibody induced recovery of the deterioration of clinical lesions as the activities of the peripheral effector T cells recovered (Fig. 8D). Subsequently, it was confirmed that the inhibitory effect on mast cell degranulation activity in ear tissue, caused by M-MSC administration, was further exacerbated by TGF-β mAb administration (Fig. 8E,F). Taken together, we confirmed the TGF-β dependence of the inhibitory effects of M-MSC administration in the CU mouse model.

## Discussion

CU is characterised by urticarial wheals and redness reactions at the site of contact with the allergen. ICU has more serious consequences than NICU, so accurate diagnosis and treatment are important. ICU is usually classified as an IgE-dependent response^1^ and is well known as a systemic inflammatory response in which the T-cell-mediated cellular immune responses are involved during onset of the disease^6–8^. These urticarial reactions progress from local wheals and redness reactions to systemic urticaria^9^. The causative agents of ICU are diverse, and it is difficult to establish a causal relationship owing to the complex pathological mechanism, which often requires long-term treatment. However, currently used treatments for urticaria have limitations, such as temporary symptom relief and specific side effects, restricting their long-term use^10–13^. Based on recent studies, a treatment proposal using stem cells has been in the spotlight as an approach for previously intractable diseases^14,15^. In particular, many studies have reported that MSCs can be usefully applied to the treatment of allergic diseases owing to their excellent immunomodulatory abilities^48–51^. However, among the various allergic prognosis, research on stem cell therapy for CU, which is known as an intractable condition, is still insufficient. In particular, the mechanism of interaction between the MSCs and effector cells in the pathogenesis of urticaria has not been reported.

We aimed to establish the basis for using M-MSCs as cell therapy agents by investigating their anti-inflammatory function. To evaluate the therapeutic effect of M-MSC administration, we established a TMA-induced CU animal model and designed various studies by varying the number of M-MSC administered under single or repeated administration conditions. In the subcutaneous single administration group, 1.0 × 10^4^ M-MSCs were administered, which had the most significant suppression effect in vivo based on the ear swelling, skin lesion formation, and itching results according to disease development (Fig. 1).

The CU mouse animal model is characterized by cell-mediated immune responses that evolve with the development of the disease. This results in a complex immune response marked by the secretion of both T_H_1- and T_H_2-derived inflammatory cytokines^7,8^. Therefore, the disease-modifying effects of M-MSC administration were investigated for control over the activities of the effector T cells by the peripherally introduced M-MSCs. As a result, M-MSC administration not only significantly improved the disease but also directly regulated the activities of effector T cells in lymph or peripheral tissues (Fig. 2A,B). The mRNA expression of major inflammatory cytokines in the ear tissue was significantly reduced through M-MSC administration, and notably, the 10 K (1.0 × 104) M-MSCs group exhibited the most effective inhibitory effect compared to other treatment groups in the experimental animals (Fig. 1G). Despite anticipating a cumulative anti-inflammatory effect through repeated M-MSC administration, no additional anti-inflammatory effect was observed in the contact urticarial model (Fig. 3). Despite the absence of standardized guidelines for stem cell administration in this animal models, we thoroughly reviewed analogous studies for guidance. However, due to the extensive variability in dosage criteria, we opted to assess multiple concentrations (5 k, 10 k, 20 k, 50 k, and 100 k) in our pilot study. Interestingly, administration of high doses of M-MSCs (50 K and 100 K) did not produce significant therapeutic effects, whereas discernible inhibitory effects were observed with lower cell doses. Considering this trend, we took into account the fact that, despite the anti-inflammatory effects in experimental animal models, stem cells inherently contain multiple antigens. The administration of an excess of stem cells, including various non-self antigens, could be conceptualized as the introduction of antigens that need to be removed within the mouse.

Consequently, in this study, we successfully replicated the reductions in ear thickness and skin lesions demonstrated by the M-MSC 5 k, 10 k, and 20 k administration groups (Figs. 1 and 2). Although a pronounced dose-dependent inhibitory effect was not evident, consistent findings led us to conclude that the 10 k dosage represented the most appropriate condition. Unlike chemical reagents or single molecules, the administration of biological entities such as cells, which inherently contain numerous antigens, may involve compatibility with the immunogenicity of the host. Despite the anti-inflammatory properties of cell therapy, it has been confirmed that excessive administration does not achieve the expected effect.

MSCs have been known to be a relatively safe and effective cell therapy for various immune diseases in the past, such as autoimmune and allergic diseases^18–26^. In this study, we compared embryonic stem cell-derived M-MSCs with bone marrow-derived BM-MSCs (a traditional source of MSCs) and a group treated with an oral antihistamine (cetirizine), which served as a positive control. We assessed the administration of M-MSCs as a significantly meaningful treatment in comparison to other positive controls using a disease animal model. The efficacy of M-MSC administration was consistently verified, and we evaluated the relative disease inhibitory effects of M-MSCs compared to the positive control group. Analysis of the results, including ear swelling, skin lesion formation, and itching, confirmed that M-MSCs produced similar or superior effects compared to the positive control groups (Fig. 4).

We confirmed the control of inflammatory responses through the inhibitory effects of M-MSC administration in contact urticaria lesions through a mechanism study. To assess the responses of M-MSCs to the activity of mast cells, the primary innate immune cells involved in the initiation of contact urticarial lesions^1^, and T cells, the key targets for cell-mediated immune responses in disease development^6–8^. After in vitro co-culture of splenic T cells and mast cells with M-MSCs, we analyzed the inhibitory effects on each type of immune cell. T cells were assessed for their control over the secretion of inflammatory cytokines by polarized effector T cells and IgE-mediated degranulation of mast cells. Co-culture with M-MSCs resulted in decreased activity of each T cell subset and limited degranulation responses of the mast cells (Fig. 5). Therefore, we confirmed that M-MSCs inhibit the development of contact urticaria by restraining the inflammatory response of effector cells.

Additionally, it is necessary to evaluate the mechanism by which the anti-inflammatory effects of M-MSCs are expressed. From past studies, it is well known that MSCs can help overcome inflammatory immune diseases through the secretion of several immunomodulatory molecules^47^. We confirmed the expressions of known candidate immunomodulatory factors to analyse which of them were activated when M-MSCs come into contact with the effector cells. The candidate genes that are expressed in human M-MSCs in a co-culture were compared, and we confirmed that the mRNA expression of TGF-β significantly increased in a co-culture with splenic lymphocytes and mast cells (Fig. 6). We hypothesised that TGF-β having homology in mice could play a role as an important regulatory molecule and confirmed the TGF-β mAb neutralization experiments in vitro (Fig. 7) and in vivo (Fig. 8). When TGF-β was neutralized, the effects of M-MSCs, including the inhibition of inflammatory cytokine secretion by effector T cell subsets and the suppression of IgE-mediated mast cell degranulation, were partially restored. Especially in the contact urticaria mouse model, M-MSC-mediated skin lesion improvement (Fig. 8A–C) and peripheral T cell activity limitation recovery were also achieved (Fig. 8D) by TGF-β mAb neutralization.

In this study, based on the significant inhibitory effects observed in a contact urticaria mouse model through the administration of M-MSCs, it was demonstrated that demonstrated a noteworthy inhibitory impact on effector T cell subsets (T_H_1, T_H_2, and cytotoxic T cells) and mast cells within an in vitro. This observation underscored a discernible TGF-β-dependent trend. Nevertheless, the regulatory effects of M-MSCs in contact urticaria are speculated to be controllable through more diverse pathways. For instance, while our study successfully directly regulated the IgE-mediated degranulation in mast cells, an important avenue for further exploration lies in assessing the indirect modulation of the IgE production pathway. Specifically, evaluating whether M-MSCs exert an indirect control over this pathway by influencing IL-13^+^ follicular helper T cells (Tfh13) at the lymphoid organ is of significant importance. Tfh13 cells have the capacity to induce the affinity maturation of IgE-producing B cells at a higher hierarchical level^52^. Given the known role of TGF-β in controlling the accumulation and effector mechanisms of Tfh cells^53^, this avenue appears promising. Furthermore, it is pertinent to note that TGF-β serves as a pivotal regulator of induced regulatory T cells (iTregs), with these mechanisms extending their influence to the regulation of mast cell activity^54^. Although our current investigation predominantly concentrated on the direct control of effector T cell subsets and mast cells, unraveling the indirect immunoregulatory mechanisms of M-MSCs through subsequent research endeavors holds substantial promise and significance.

## Conclusion

The administration of M-MSCs in a contact urticaria mouse model showed improved severity and symptoms of the disease, yielding more effective therapeutic outcomes compared to treatment with BM-MSCs or cetirizine. Among various factors derived from M-MSCs, TGF-β exhibited specific activation in co-culture with effector cells such as T cells and mast cells. Through TGF-β neutralization studies, we confirmed the potential of M-MSCs to regulate effector cells, including T cells and mast cells, in a TGF-β-dependent manner. While more in-depth clinical and multi-mechanism studies are necessary, these findings provide a basis for utilizing M-MSCs in novel therapeutic strategies for contact urticaria.

### Supplementary Information

Supplementary Information.

## Data Availability

The datasets used and/or analyzed during the current study are available from the corresponding authors on reasonable request.

## Abbreviations

CU: Contact urticarial
TMA: Trimellitic anhydride
IgE: Immunoglobulin E
FceRI: High-affinity IgE receptor
M-MSCs: Multipotent-mesenchymal stem cells
cLN: Cervical lymph node
IL-4: Interleukin-4
IL-6: Interleukin-6
IFN-γ: Interferon-γ
TNF-α: Tumor necrosis factor-α
HGF: Hepatocyte growth factor
IDO-1: Indoleamine 2,3-dioxygenase-1
PD-L1: Programmed death-1 ligand
IL-10: Interleukin-10
PGE2: Prostaglandin-E2
TGF-β: Transforming growth factor-β
