# Supplementary material for: Embryonic-stem-cell-derived mesenchymal stem cells relieve experimental contact urticaria by regulating the functions of mast cells and T cells
Source: Sci Rep. 2023 Dec 20;13:22694. doi: 10.1038/s41598-023-50258-2 (PMC10733409; doi:10.1038/s41598-023-50258-2)
Supplement: Supplementary file 1 — Supplementary Information. [file 41598_2023_50258_MOESM1_ESM.pdf]

Embryonic-stem-cell-derived mesenchymal stem cells relieve experimental contact urticaria by regulating the functions of mast cells and T cells

Seung Yeun Hyun[1†], Eun-Young Kim[2†], Minseong Kang[1], Jeong Won Park[1], Ki-Sung Hong[2], Hyung-Min Chung[2,3], Wahn Soo Choi[3], Se-Pill Park[2,4*], Geunwoong Noh[5*] and Hyuk Soon Kim[1*]

[1]Department of Biomedical Sciences, College of Natural Science and Department of Health Sciences, The Graduate School of Dong-A University, Busan 49315, Korea, [2]Miraecellbio Co., Ltd., Seoul 04795, Korea, [3]School of Medicine, Konkuk University, Seoul 05029, Korea, [4]Department of Bio Medical Informatics, College of Applied Life Sciences, Jeju National University, Jeju 63243, Korea. [5]Department of Allergy, Allergy and Clinical Immunology Center, Cheju Halla General Hostpital, Jeju 63127, Korea.

[†]Seung Yeun Hyun and Eun-Young Kim contributed equally to this work.

*Correspondence: hskimxo@dau.ac.kr; sppark@jejunu.ac.kr; admyth@naver.com

Supplementary Figures

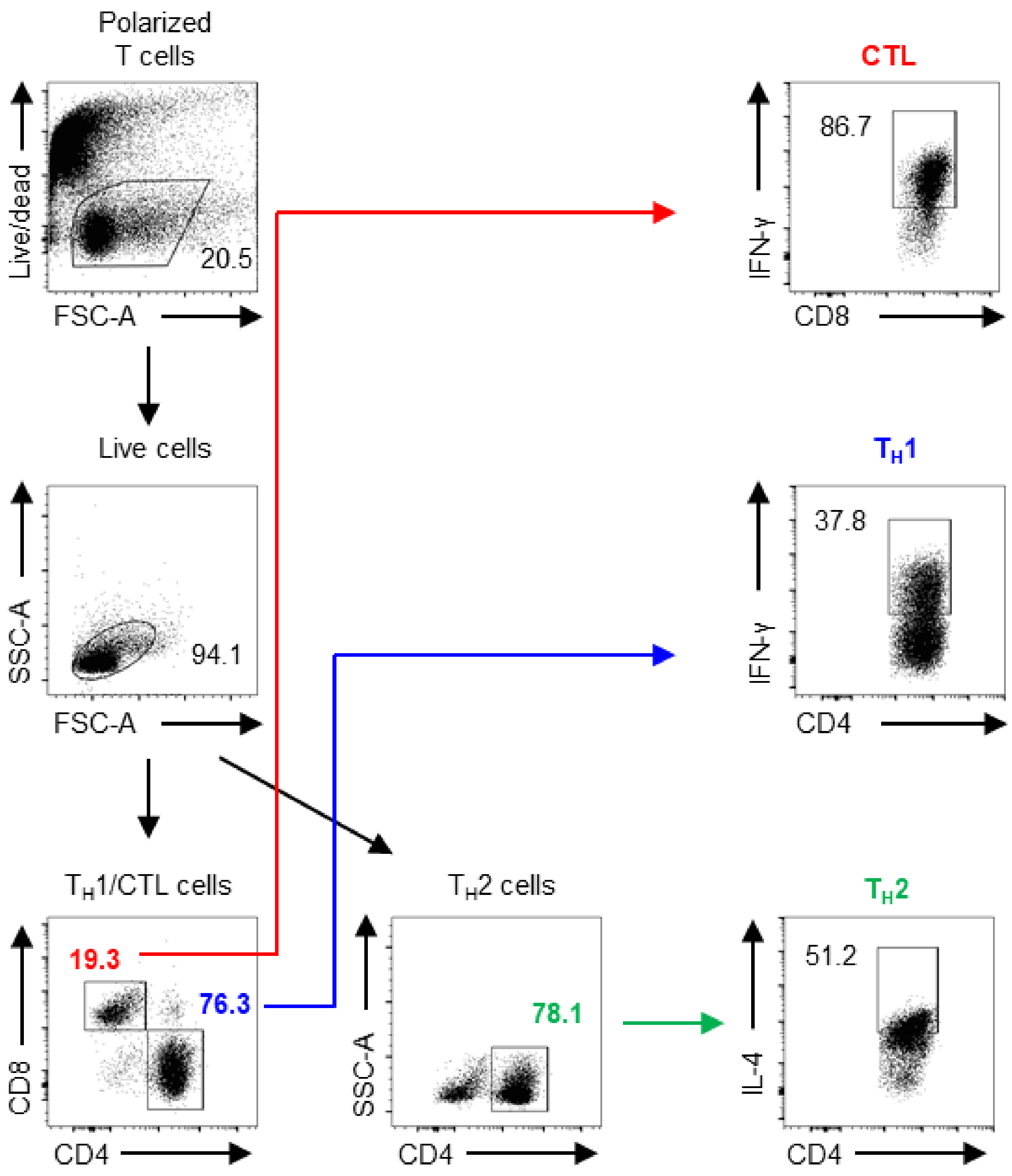

Supplementary Figure 1. Specific gating strategies diagram of flow cytometry for T subsets.

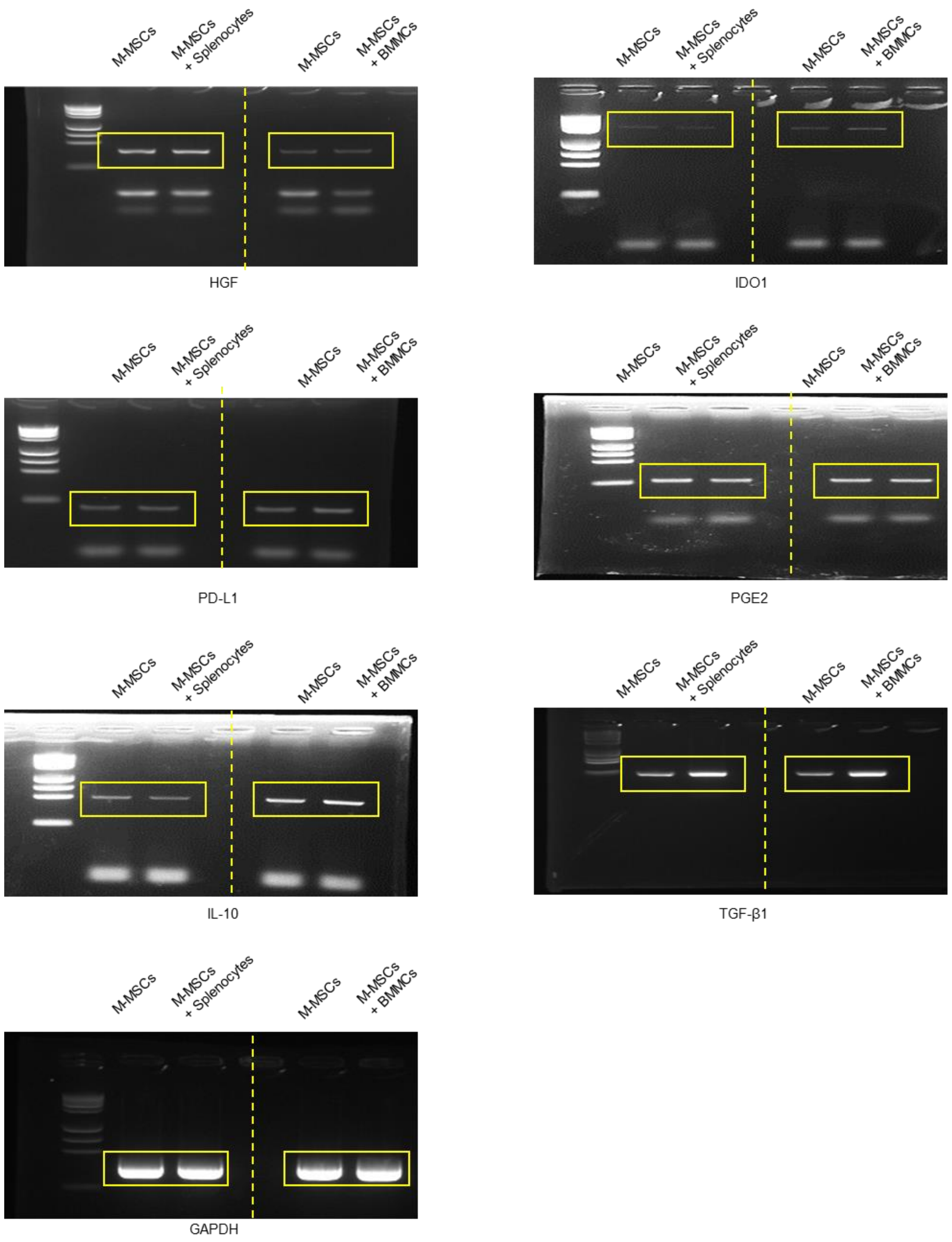

Supplementary Figure 2. Uncropped gel images of electorphoresis data for figure 6A and 6Band B.

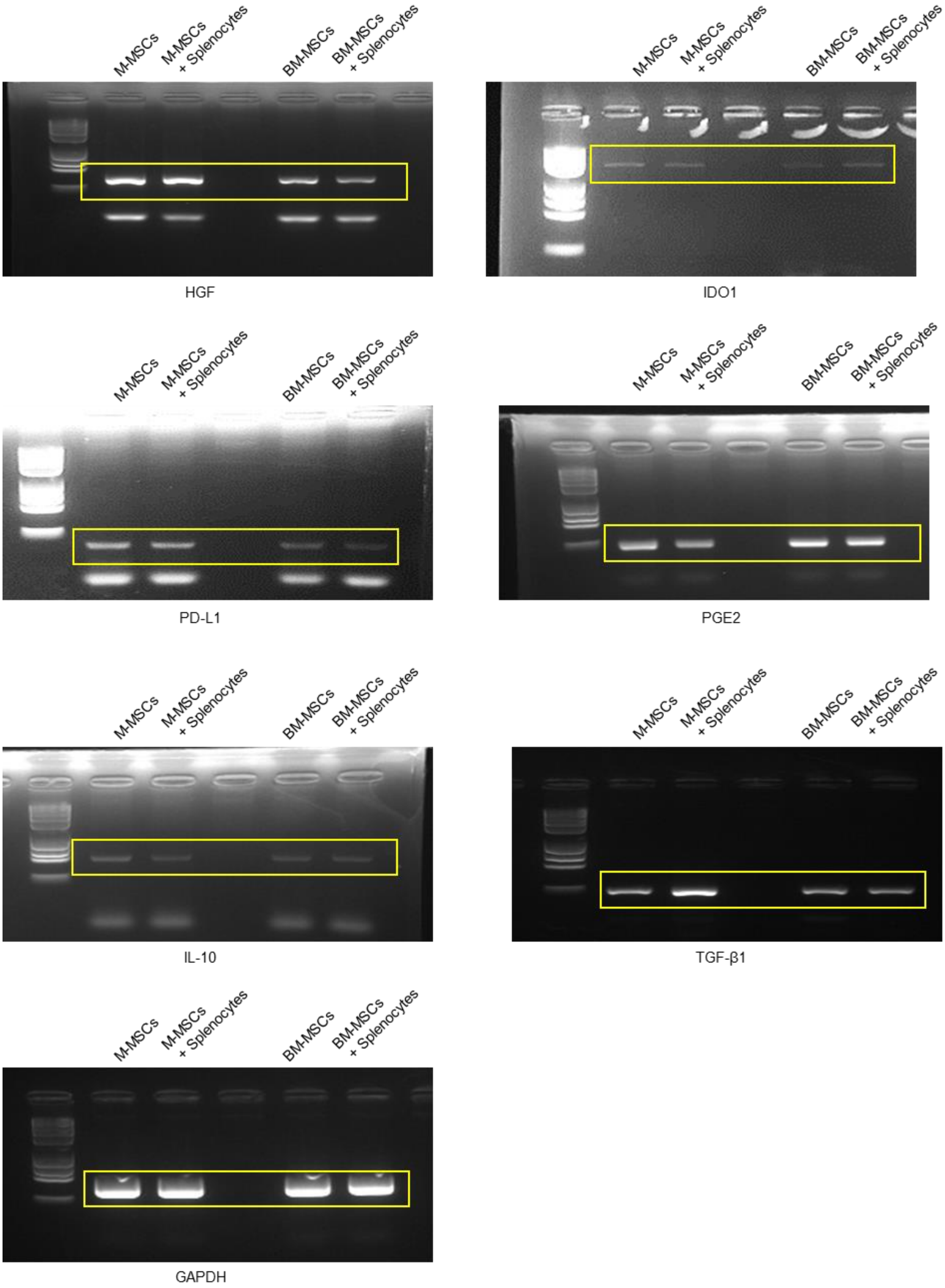

Supplementary Figure 3. Uncropped gel images of electorphoresis data for figure 6C

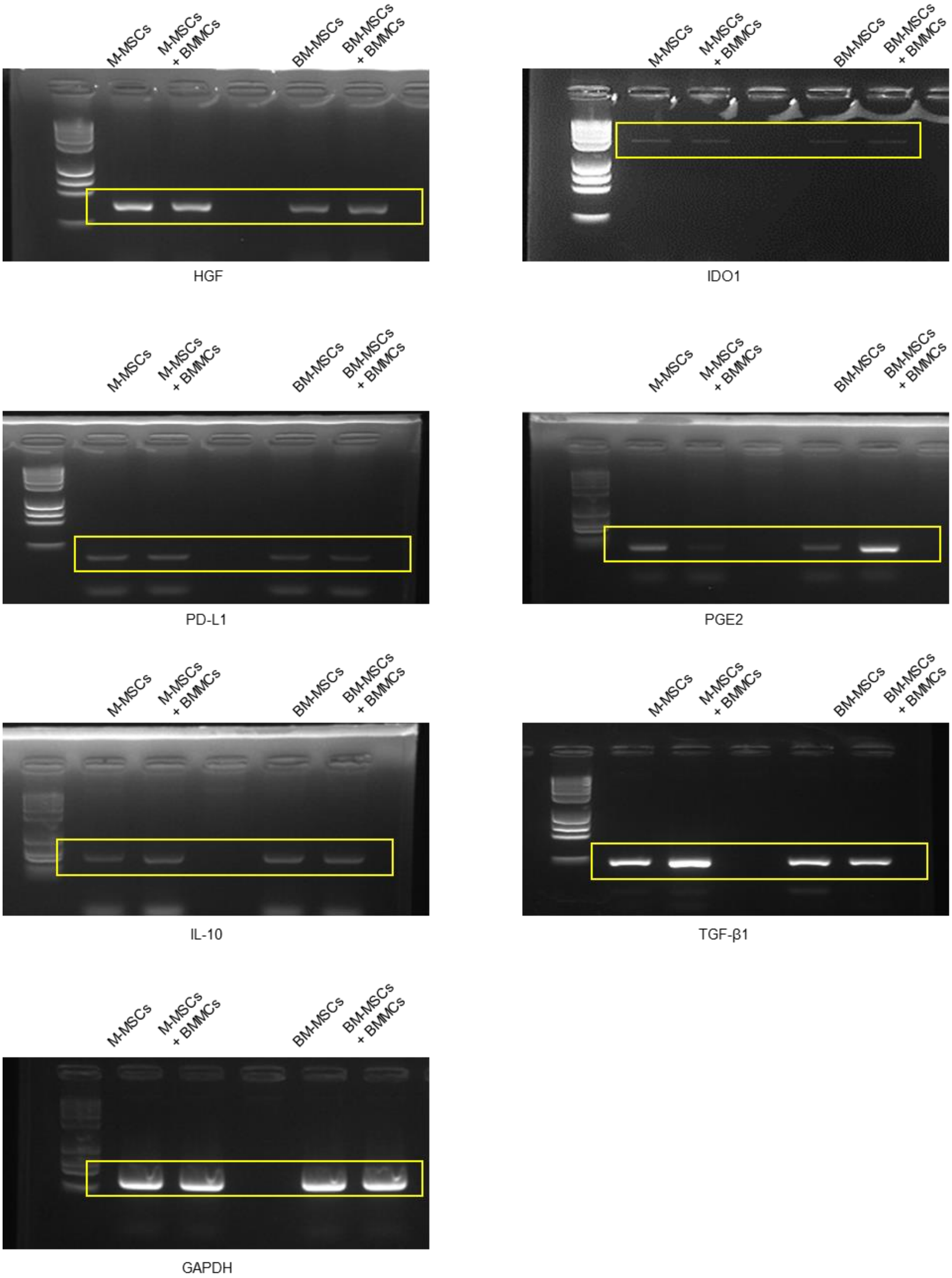

Supplementary Figure 4. Uncropped gel images of electorphoresis data for figure 6D
